# Transanal proctocolectomy and ileal pouch-anal anastomosis (TaIPAA) for ulcerative colitis: medium term functional outcomes in a single centre

**DOI:** 10.1186/s12893-020-01007-z

**Published:** 2021-01-06

**Authors:** G. T. Capolupo, F. Carannante, G. Mascianà, S. Lauricella, E. Mazzotta, M. Caricato

**Affiliations:** Department of Colorectal Surgery, Campus Bio-Medico University of Rome, Via Alvaro del Portillo 21, 00128 Rome, Italy

**Keywords:** TaIPAA, Proctocolectomy, Colorectal surgery, IBD, Ulcerative colitis, Laparoscopic surgery

## Abstract

**Background:**

Transanal dissection of the rectum has been recently introduced for ileal pouch-anal anastomosis (IPAA) in UC showing promising results. Thanks to the precise identification of the rectotomy site the risk of long rectal stump is avoided, and a single stapled anastomosis is performed easily. The aim of this study is to analyze our initial experience of transanal proctocolectomy and ileal pouch-anal anastomosis (TaIPAA), considering postoperative complications and medium-term functional outcomes.

**Methods:**

Our Center has experienced the transanal approach for proctectomy and IPAA since August 2018. All patients underwent Enhanced Recovery After Surgery (ERAS) protocol. Postoperative complications occurring within 30 days after surgery were taken into consideration. Fecal continence, genito-urinary activity and global quality of life at 1 and 6 months after ileostomy reversal have been assessed.

**Results:**

Until March 2019, 8 patients underwent transanal proctocolectomy and ileal pouch-anal anastomosis (TaIPAA). In all cases the laparoscopic approach was performed during the transabdominal phase; abdominal drainage was never used. At the time of the pouch construction a defunctioning loop ileostomy was created in all patients. Stoma closure was performed in all cases at a median time of 6 months after surgery. Postoperative complications occurred in only one patient, who showed rectal bleeding, not required a re-invertation. There were no cases of anastomotic leakage. Medium-term functional outcomes were determined prospectively using previously validated quality of life questionnaires (Cleveland Global Quality of Life). Fecal incontinence for liquid or solid stool, genitourinary and sexual functions were also investigated, showing comparable results with the literature data.

**Conclusions:**

In our experience, transanal proctocolectomy and ileal pouch-anal anastomosis provided good short and medium-term functional results in UC.

## Background

Restorative proctocolectomy is widely adopted in the treatment of ulcerative colitis (UC) [1–3] as well as in other inflammatory and neoplastic conditions, requiring an ileal pouch-anal anastomosis (IPAA) to reconstruct gastrointestinal continuity to the anus [4].

Conventionally, either the laparoscopic or the open approach can be employed to gain rectal dissection and creation of ileal pouch-anal anastomosis. Pouch-anal anastomosis is usually made using a stapler, leaving a 2 cm rectal cuff in order to preserve continence and to reduce the risk of inflammatory recurrence or dysplasia [5]. The dissection of the last centimeters of the rectum, rectum resection and ileal pouch-anal anastomosis could be demanding from a technical point of view due to narrow pelvic space and cross stapling of the distal part of the rectum is often challenging for surgeons.

Transanal total mesorectal excision (TaTME) has been recently described in rectal cancer treatment [6], with potential technical and oncologic advantages compared to transabdominal approach [7], especially in man.

The transanal approach for the proctectomy has been described also in IPAA since 2015 [8], showing feasibility and potential technical advantages; some series [9–12] and initial comparative studies have been published [13, 14], showing a similar rate of postoperative morbidity, equivalent quality of life and functional results. The complete mesorectal excision is not required for this indication and is rarely performed in the described experiences.

The aim of our study is to analyze a single Centre experience of trans-anal IPAA (Ta-IPAA), examining early postoperative complications, medium-term functional outcomes and quality of life.

## Methods

In our Center the trans-anal approach for restorative proctocolectomy has been performed since August 2018. All epidemiological data were extrapolated from the hospital information system; a unique IBD database was set up. Baseline epidemiological characteristics of the patients were examined; the Mayo Score for UC was used to define the disease activity index [15]. Enhanced Recovery After Surgery (ERAS) protocol was applied to all patients. Postoperative complications where classified according to Clavien–Dindo [16]. Preliminary functional outcomes were assessed in the outpatient clinic, from 1 to 6 months after ileostomy reversal, or 7 to 12 months from the time of the Ta-IPAA, with the use of validated questionnaires. Fecal incontinence for liquid or solid stool, restriction in work and social life were investigated with the use of the Wexner Continence Grading Scale [17] at 1 and 6 mo. after ileostomy reversal. Genito-urinary symptoms have been investigated with a gender-specific questionnaire 6 mo. after ileostomy reversal: International Erectile Function Score (IEFS-5) [18] for males and Female Sexual Function Index (FSFI) [19] for females. Quality of life was evaluated at 6 mo. after ileostomy closure according to Fazio et al. [20], using both the validated Cleveland Global Quality of Life (CGQL) questionnaire, and the happiness with surgery.

The Cleveland Quality of Life Score (Fazio score) [20] is a simple comprehensive score specifically designed for pelvic pouch surgery patients, investigating three items: the quality of life, quality of health and energy level. Each item is graded on a scale of 0–10 (where 10 is the best). The three scores were added, and the final CGQL utility score was calculated dividing this result by thirty [20, 21], happiness with surgery is a simple scale, evaluated with a score from 0 to 10, with 10 representing complete satisfaction with the procedure, already described by Fazio 1999 [20].

### Surgical technique

A detailed description about the surgical technique and its variations is reported in the paper from Borja de Lacy et al. [22] Here we report a summary of the surgical procedure. The operation can be performed in two-steps (first step: subtotal colectomy and trans-anal proctectomy, pouch construction and IPAA with diverting stoma; second step: stoma closure) or three-steps (first step: subtotal colectomy with end-ileostomy and no proctectomy; second step: trans-anal proctectomy, pouch construction, IPAA and diverting stoma; third step; stoma closure) according to the clinical conditions and choice of the patient [23–25].

The abdominal phase can be performed using the laparoscopic or the open approach. The trans-anal phase starts with the introduction of a GelPoint Path® (Applied Medical, Rancho S. Margarita CA USA) with three working Ports and a standard 10 mms 30° laparoscopic camera to access the rectal lumen. A 0 Polypropylene purse-string suture is placed for rectal lumen closure, 4 cm above the dentate line, carbon dioxide is insufflated at a pressure of 12 mmHg to distend the rectal stump and the dissection field, and the proctotomy is started 1 cm distally. This allows the surgeon to leave a rectal cuff about 2 cms, which is related to less recurrence or neoplastic risk.

The purse-string suture avoids that the insufflation in the rectal stump distend the whole colon and facilitates dissection and hemostasis. The specimen quality is not a performance indicator in proctectomy for UC, giving the chance to make close rectal dissection or intra-mesorectal dissection close to the bowel wall [26]. This latest approach has the potential advantage to reduce the risk of pelvic nerve and urethral injury but could cause a more relevant blood loss. In our experience the rectal dissection has always been performed in the intra-mesorectal plane.

Transanal dissection continues until the rectal surgeon “meets” the abdominal surgeon from the opposite direction. The specimen should be brought out through the Pfannenstiel mini laparotomy, trans-anally or through the pre-existing or future stoma site, according to the surgeon evaluation. In all cases, the wound is protected with a wound protector (Alexis®, Applied Medical, Rancho S. Margarita CA USA) which enables a laparoscopic approach trough the extraction site before and after specimen retrieval, and is useful to prevent SSI [27]. A 12 cm long J-pouch is created extracorporeally with two loops of the small bowel, using a linear stapler. Another 0 Polypropylene purse-string suture is hooked on the distal rectal cuff. The end-to-end ileal pouch-anal anastomosis is created with a single circular stapler. The integrity of the anastomosis is evaluated through the reverse air leak test and ICG angiography can be performed to assess adequate mucosal and anastomotic perfusion. A loop ileostomy is created with the skin-bridge technique [28]. The stoma closure represents the last phase of surgical treatment and it is performed after endoscopic evaluation of the pouch and pouch-anal anastomosis. All patients were enrolled in enhanced recovery after surgery (ERAS) protocol [29].

## Results

Between October 2018 and March 2019, in our Colorectal Surgery Unit, 8 patients affected by UC underwent Ta-IPAA.

The median age was 54 years (range 28–79) Six patients presented at the operation with moderate disease (6–10 Mayo score). Two patients presented with severe disease and required urgent surgery (Table 1); one of them presented with toxic mega colon.

**Table 1:** Clinical details at baseline

Patient	Sex	Age	BMI	ASA	Mayo score
1	1	50–60	20–25	3	8
2	1	70–80	20–25	3	9
3	2	50–60	30–35	3	9
4	1	60–70	20–25	3	10
5	1	20–30	20–25	2	9
6	2	50–60	25–30	2	12
7	2	50–60	20–25	2	12
8	1	50–60	25–30	2	9

In all cases the multi-portal laparoscopic approach was employed to achieve the subtotal colectomy. We had no cases of conversion from laparoscopy to open surgery. Six patients underwent three-steps and two patients two-steps procedure. The waiting time from one step to the other was dictated by the clinical conditions of the patient, ranging from 1 to 8 mo. The specimen was brought out through the Pfannenstiel mini laparotomy or through the stoma site in a single case. All patients had a single circular-stapled ileo-anal pouch anastomosis as described above. All patients received a defunctioning skin bridge loop right ileostomy (Table 2).

**Table 2:** Surgical details and operative time of proctectomy and IPAA

Patient	Operation	Conversion	Anastomosis	Diverting stoma	Operating time (mins): II surgical stage (Proctectomy + IPAA)	Operating time (mins): I surgical stage (Colectomy + proctectomy + IPAA)
1	Three-stage RPC	No	Circular Stapler	Yes	294	
2	Three-stage RPC	No	Circular Stapler	Yes	282	
3	Two-stage RPC	No	Circular Stapler	Yes		354
4	Three-stage RPC	No	Circular Stapler	Yes	282	
5	Three-stage RPC	No	Circular Stapler	Yes	258	
6	Three-stage RPC	No	Circular Stapler	Yes	354	
7	Three-stage RPC	No	Circular Stapler	Yes	299	
8	Two-stage RPC	No	Circular Stapler	Yes		489

For patients operated in II stages, the operating time includes the colectomy. Three-stage restorative proctocolectomy (RPC): [subtotal colectomy + proctectomy and ileal pouch-anal anastomosis (IPAA) + closure of ileostomy], Two-stage RPC (total proctocolectomy and IPAA + closure of ileostomy), *NA* not applicable

### Early post-operative course

According to Clavien–Dindo classification, postoperative complications occurring within 30 days after surgery are reported. One patient had urinary infection treated with antibiotic therapy after the second phase of surgery (Clavien–Dindo Grade II). One patient had ileus after both operations; fasting was the only therapy (Clavien–Dindo Grade I). One patient, presented with toxic megacolon at the operation, had pulmonary infection (Clavien–Dindo Grade II). Only one patient required surgical intervention for a large parastomal hernia seven days after surgery (Clavien–Dindo Grade IIIb) (Table 3).

**Table 3:** Post-operative complications (recorded and classified according to Clavien – Dindo [16])

Patient	Operation	1st step surgery Clavien–Dindo	2nd step surgery Clavien–Dindo	Stoma reversal
1	Three-stage RPC	–	II	–
2	Three-stage RPC	–	IIIb	–
3	Two-stage RPC	–	NA	–
4	Three-stage RPC	–	–	–
5	Three-stage RPC	I	I	–
6	Three-stage RPC	–	–	–
7	Three-stage RPC	II	–	–
8	Two-stage RPC	–	NA	–

Three-stage restorative proctocolectomy (RPC): [subtotal colectomy + proctectomy and ileal pouch-anal anastomosis (IPAA) + closure of ileostomy]; Two-stage RPC (total proctocolectomy and IPAA + closure of ileostomy); *NA* not applicable

No cases of anastomotic leakage were found. Only one case developed a surgical site infection (SSI) graded Clavien Dindo II after the II surgical stage.

The median Length of Stay (LOS) for the first phase of the three-stage surgery was 6,8 days (range 3–16). The LOS for the second phase of the three-stage surgery was 7 days (range 2–14). The LOS for the restorative proctocolectomy in two step surgery was 5,5 days. Pathological examination showed low-grade dysplasia in all patients; IntraEpithelial Neoplasia was never found. The loop ileostomy was closed at a median time of 6 months after RPC, according to the institutional protocol and caseload, following an endoscopic examination and an X-ray pouchgraphy. The endoscopic evaluation of the rectal cuff showed no variability, with a length of 2 cm. in all cases. Loop ileostomy take-down was performed with the purse-string technique and we report no postoperative complications. Length-of-stay after stoma closure was 4 ± 2 days. The results of Quality of Life questionnaire are reported in Table 4. Table 5 shows the details of Cleveland Quality of Life Score. Happiness-with-surgery [20] scores ranged from 7 to 10 (median 8).

**Table 4:** Patient reported outcomes

Patient	Sex	WCGS 1 mo	WCGS 6 mo	IIEF	FSFI	CGQLS
1	M	5/20	4/20	27/30		0.56
2	M	3/20	3/20	0/30		0.70
3	F	1/20	0/20		26/36	0.53
4	M	4/20	4/20	30/30		0.66
5	M	3/20	1/20	28/30		0.63
6	F	0/20	0/20		30/36	0.73
7	F	1/20	0/20		28/36	0.70
8	M	2/20	1/20	30/30		0.66

*WCGS* Wexner continence grading scale, *IIEF* International Erectile Function Score, *FSFI* female sexual function index, *CGQLS* Cleveland Global Quality of Life Scale

**Table 5:** Details of Cleveland Global Quality of Life

Patient	Quality of life	The quality of health	Energy level	Final score
1	7	5	5	0.56
2	7	6	8	0.70
3	4	8	4	0.53
4	7	7	6	0.66
5	7	6	6	0.63
6	8	7	7	0.73
7	8	7	6	0.70
8	7	7	6	0.66

The CGQL is a simple comprehensive score specifically designed for pelvic pouch surgery patients, investigating three items: the quality of life, quality of health and energy level. Each item is graded on a scale of 0–10 (where 10 is the best). The three scores were added and the final CGQL utility score was calculated dividing this result by thirty, giving a result ranged from 0 to 1

## Discussion

Ileal-pouch-anal anastomosis (IPAA) represents the standard of care in patients who underwent total proctocolectomy for Ulcerative Colitis (UC). The technique has evolved from the initial description by Sir Alan Parks and J. Nicholls in 1978 [4]. Several pouch designs have been described, but J pouch allows a shorter operating time with equivalent long-term results [30]. Feasibility and safety of laparoscopic colectomy and pouch construction and IPAA have been clearly demonstrated, and data published in 2011 has shown a reduction in complications rate after laparoscopic surgery [31]. The anastomosis can be double-stapled, requesting the preservation of a segment of distal rectum, or mucosectomy and hand sewn anastomosis can be performed, allowing a complete removal of the affected rectal mucosa. The latter option shows a poorer continence and lower anal resting pressure [32]. A systematic review [33] shows that the risk of high-grade dysplasia is quite low (0,15%) after restorative proctocolectomy and that the frequency is the same in the pouch, anal transitional zone and rectal cuff. Stapled IPAA is currently considered the standard of care [34]; mucosectomy and hand-sewn pouch-anal anastomosis are reserved mostly to the failed stapled anastomoses and represent less than 5% of the procedures, despite the lack of a clear evidence against its use [5].

With the use of a stapled anastomosis, the length of the rectal cuff is one major determinant of quality of life after operation. A cuff length > 2 cm is related to symptomatic inflammatory disease recurrence or neoplastic risk.

The trans-anal approach can be employed, both in cancer and IBD surgery, to overcome some limitations of the traditional minimally invasive techniques, thus allowing better visualization in the low pelvis and easier dissection of the distal 5 cm of the rectum. It has been introduced in the last 10 years and rapidly gained a widespread adoption, promising to overcome the limitations of laparoscopic rectal cancer surgery. Recent concerns have been raised on short-term oncological outcomes leading to a regional moratorium in Norway [35]. First functional data in a series of TaTME performed for rectal cancer showed preserved urinary and sexual function and low incidence and severity of low anterior resection syndrome (LARS).

The trans-anal approach represents the newest option for restorative proctocolectomy (RPC), improving the technical steps of a complex operation and possibly the surgical outcomes. In IBD patients, oncologic outcomes do not apply, while health-related quality of life for this type of patients is very important, considering their young age and life expectancy.

The technique was first described in human by the Barcelona team in 2015 after the initial animal and cadaveric experiences, with a first case series involving 16 cases [8]. Since then, we found in literature 5 single-case reports [36–40], 4 further case series ranging from 8 to 18 cases [9–12] and three multicentric experiences, reporting partially overlapping cohorts of 97 [13], 62 [41] and 100 [14] cases. Described cases vary in indication from IBD to FAP.

Some initial quality of life evaluation reports comparable results, with some experiences showing slightly higher satisfaction with urinary status [42] and others reporting slightly worse results for fecal incontinence [43].

Reported experiences agree that Ta-IPAA allows a better visualization of the distal 5 cm of rectum, making easier the identification of a < 2 cm rectal cuff and a more precise pelvic dissection.

To achieve a 2 cm rectal cuff, with the trans-anal approach we make a purse-string 4 cm proximal to the dentate line and make a rectotomy 1 cm distally. A further cm of rectal wall will be removed in the stapler doughnut, thus leaving the correct length of rectal stump, allowing a low risk of symptomatic disease and an optimal conservation of the anal sensitivity for a better continence.

Moreover, Ta-IPAA permits the identification of the site for rectal section and the realization of a trans-anal distal purse-string for the stapled anastomosis, avoiding multiple stapler firings for rectal stump closure and lowering the risk of anastomotic leakage [44].

This technique in IBD treatment was employed only by few expert surgeons; moreover, solid data concerning long term functional outcomes are still restricted. De Buck van Overstraeten A. et al. [13] published short-term outcomes of 97 patients from 3 Institutions who underwent a single incision surgery combined with TaTME for ileoanal pouch construction, compared with 119 cases in which a transabdominal approach was employed. They demonstrated the safety of Ta-IPAA for UC and showed a lower rate of 90 days postoperative complications in ulcerative colitis, comparing Ta-IPAA to transabdominal approach. Short-term complications vary widely across the different experiences: a very large experience form Cleveland Clinic (Cleveland OH, USA) reported a short-term pelvic sepsis rate of 6.5%, and an anastomotic leak rate of 4.8% [20].

A more recent paper reported a multicentric experience evaluating the long-term outcomes of Ta-IPAA against transabdominal-IPAA in restorative proctocolectomy, in 100 vs 274 cases. It shows that severe complication rate was significantly reduced, whereas anastomotic leak rate was non significantly lower with Ta-IPAA. The severity of clinical presentation and the possible presence of toxic megacolon has not a direct impact to the technique: more severe cases are treated in three stages, separating the critical phase of the disease from the pelvic procedure.

Quality of life and functional outcome are crucial issues in restorative proctocolectomy, and current results, although encouraging, report a rate of pouch failure up to 20% in 20 years’ experience [45]. Technical refinements are therefore required in order to achieve better functional results. Many scales have been proposed to evaluate functional aspects in the main areas of continence, genito-urinary functions and general quality of life. In our initial experience we employed very simple, easy to administer questionnaires, in order to obtain a simple evaluation of the most important domains. The Wexner Continence Grading Scale [17] is an easy to administer, widely used questionnaire that investigate type and frequency of incontinence episodes. The same experience including 1156 IPAA patients reports in the 1st year after surgery a mean CGQL score of 0.80 (95% CI 0.78 to 0.81), higher than in our experience. The cutoff for sexual dysfunction for women has been determined in 26,55 [46] and our results show that all women are around or over the threshold; the cutoff for good erectile function is considered to be 24 [47] and our population has a score higher for all cases other than one with age over 70. The majority of IPAA patients shows some degree of incontinence: in a Norwegian experience [48] the mean score is reported to be 7,8 (range 0–17), higher for patients with hand-sewn anastomosis, in our experience the incontinence score is lower both at 1 and 6 mo. In the paper from Chandrasinghe et al. [14] no statistically significant differences between Ta-IPAA and transabdominal-IPAA are reported considering quality of life, and Ta-IPAA is associated with higher quality of health and energy level.

Similarly, to what happens for many complex surgical procedures, the surgeon and center caseload have a significant impact on postoperative morbidity and mortality, making difficult results comparison. Nevertheless, on the basis of these experiences, we can consider our results as satisfactory.

Our study presents several limitations. The first is related to the very small sample size, with a wide range of severity of the clinical presentation, which includes also one case of toxic megacolon. This is the reason we decided to make a non-comparative report. The absence of a comparative analysis makes impossible to draw any conclusion from our results, but only to confirm the feasibility of the technique, already demonstrated by others, and to point out the need or comparative studies.

## Conclusion

In conclusion, our small experience confirms preliminary data available showing that Ta-IPAA provides very good short- and medium-term functional results for ileoanal pouch surgery. The major limitation of our study is related to the small number of cases operated in a single center. More studies are needed to confirm our results.

## Data Availability

The datasets used and/or analyzed during the current study are available from the corresponding author on reasonable request.

## Abbreviations

TaIPAA: Transanal protocolectomy and ileal pouch-anal anastomosis
TaTME: Transanal total mesorectal excision
ERAS: Enhanced recovery after surgery
UC: Ulcerative colitis
RPC: Restorative proctocolectomy
RPC: Restorative proctocolectomy
IBD: Inflammatory bowel disease
CGQL: Cleveland global quality of life
SSI: Surgical site infection

## References

[1] Seah D, De Cruz P (2016). Review article: the practical management of acute severe ulcerative colitis. Aliment Pharmacol Ther.

[2] Randall J, Singh B, Warren BF, Travis SPL, Mortensen NJ, George BD (2010). Delayed surgery for acute severe colitis is associated with increased risk of postoperative complications. Br J Surg.

[3] Kornbluth A, Sachar DB (1997). Ulcerative colitis practice guidelines in adults American College of Gastroenterology, Practice Parameters Committee. Am J Gastroenterol..

[4] Parks AG, Nicholls RJ (1978). Proctocolectomy without ileostomy for ulcerative colitis. Br Med J.

[5] Øresland T, Bemelman WA, Sampietro GM, Spinelli A, Windsor A, Ferrante M (2015). European evidence based consensus on surgery for ulcerative colitis. J Crohns Colitis.

[6] Sylla P, Rattner DW, Delgado S, Lacy AM (2010). NOTES transanal rectal cancer resection using transanal endoscopic microsurgery and laparoscopic assistance. Surg Endosc.

[7] Penna M, Hompes R, Arnold S, Wynn G, Austin R, Warusavitarne J (2017). Transanal total mesorectal excision: international registry results of the first 720 cases. Ann Surg.

[8] Tasende MM, Delgado S, Jimenez M, Del Gobbo GD, Fernández-Hevia M, DeLacy B (2015). Minimal invasive surgery: NOSE and NOTES in ulcerative colitis. Surg Endosc.

[9] de Buck van Overstraeten A, Wolthuis AM, D’ Hoore A (2016). Transanal completion proctectomy after total colectomy and ileal pouch-anal anastomosis for ulcerative colitis: a modified single stapled technique. Colorectal Dis..

[10] Ambe PC, Zirngibl H, Möslein G (2017). Initial experience with taTME in patients undergoing laparoscopic restorative proctocolectomy for familial adenomatous polyposis. Tech Coloproctology.

[11] Leo CA, Samaranayake S, Perry-Woodford ZL, Vitone L, Faiz O, Hodgkinson JD (2016). Initial experience of restorative proctocolectomy for ulcerative colitis by transanal total mesorectal rectal excision and single-incision abdominal laparoscopic surgery. Colorectal.

[12] Levic Souzani K, Nielsen CB, Bulut O (2019). Transanal completion proctectomy with close rectal dissection and ileal pouch-anal anastomosis for ulcerative colitis. Asian J Endosc Surg.

[13] de Buck van Overstraeten A, Mark-Christensen A, Wasmann KA, Bastiaenen VP, Buskens CJ, Wolthuis AM (2017). Transanal versus transabdominal minimally invasive (completion) proctectomy with ileal pouch-anal anastomosis in ulcerative colitis: a comparative study. Ann Surg..

[14] Chandrasinghe P, Carvello M, Wasmann K, Foppa C, Tanis P, Perry-Woodford Z (2020). Transanal ileal pouch-anal anastomosis for ulcerative colitis has comparable long-term functional outcomes to transabdominal approach: a multicentre comparative study. J Crohns Colitis.

[15] Schroeder KW, Tremaine WJ, Ilstrup DM (1987). Coated oral 5-aminosalicylic acid therapy for mildly to moderately active ulcerative colitis. A randomized study. N Engl J Med..

[16] Dindo D, Demartines N, Clavien P-A (2004). Classification of surgical complications: a new proposal with evaluation in a cohort of 6336 patients and results of a survey. Ann Surg.

[17] Jorge JM, Wexner SD (1993). Etiology and management of fecal incontinence. Dis Colon Rectum.

[18] Rhoden EL, Telöken C, Sogari PR, Vargas Souto CA (2002). The use of the simplified International Index of Erectile Function (IIEF-5) as a diagnostic tool to study the prevalence of erectile dysfunction. Int J Impot Res.

[19] Carpenter JS, Jones SMW, Studts CR, Heiman JR, Reed SD, Newton KM (2016). Female sexual function index short version: a MsFLASH item response analysis. Arch Sex Behav.

[20] Fazio VW, O’Riordain MG, Lavery IC, Church JM, Lau P, Strong SA (1999). Long-term functional outcome and quality of life after stapled restorative proctocolectomy. Ann Surg..

[21] Coffey JC, Winter DC, Neary P, Murphy A, Redmond HP, Kirwan WO (2002). Quality of life after ileal pouch-anal anastomosis: an evaluation of diet and other factors using the Cleveland Global Quality of Life instrument. Dis Colon Rectum.

[22] de Lacy FB, Keller DS, Martin-Perez B, Emile SH, Chand M, Spinelli A (2019). The current state of the transanal approach to the ileal pouch-anal anastomosis. Surg Endosc.

[23] Hyman NH, Cataldo P, Osler T (2005). Urgent subtotal colectomy for severe inflammatory bowel disease. Dis Colon Rectum.

[24] Bitton A, Buie D, Enns R, Feagan BG, Jones JL, Marshall JK (2012). Treatment of hospitalized adult patients with severe ulcerative colitis: Toronto consensus statements. Am J Gastroenterol..

[25] Alves A, Panis Y, Bouhnik Y, Maylin V, Lavergne-Slove A, Valleur P (2003). Subtotal colectomy for severe acute colitis: a 20-year experience of a tertiary care center with an aggressive and early surgical policy. J Am Coll Surg.

[26] Carannante F, Mazzotta E, Mascianá G, Caricato M, Capolupo G. Intramesorectal or total mesorectal excision for ulcerative colitis: what is better for the patient? Minerva Chir. 2020. 10.23736/S0026-4733.20.08479-5 32975388

[27] Capolupo GT, Lauricella S, Mascianà G, Caricato C, Angeletti S, Ciccozzi M (2019). O-ring protector in prevention of ssis in laparoscopic colorectal surgery. JSLS..

[28] Carannante F, Mascianà G, Lauricella S, Caricato M, Capolupo GT (2019). Skin bridge loop stoma: outcome in 45 patients in comparison with stoma made on a plastic rod. Int J Colorectal Dis.

[29] Gustafsson UO, Scott MJ, Hubner M, Nygren J, Demartines N, Francis N (2019). Guidelines for perioperative care in elective colorectal surgery: enhanced recovery after surgery (ERAS®) society recommendations: 2018. World J Surg.

[30] McCormick PH, Guest GD, Clark AJ, Petersen D, Clark DA, Stevenson AR (2012). The ideal ileal-pouch design: a long-term randomized control trial of J- vs W-pouch construction. Dis Colon Rectum.

[31] Fleming FJ, Francone TD, Kim MJ, Gunzler D, Messing S, Monson JRT (2011). A laparoscopic approach does reduce short-term complications in patients undergoing ileal pouch-anal anastomosis. Dis Colon Rectum.

[32] Schluender SJ, Mei L, Yang H, Fleshner PR (2006). Can a meta-analysis answer the question: is mucosectomy and handsewn or double-stapled anastomosis better in ileal pouch-anal anastomosis?. Am Surg.

[33] Scarpa M, van Koperen PJ, Ubbink DT, Hommes DW, ten Kate FJW, Bemelman WA (2007). Systematic review of dysplasia after restorative proctocolectomy for ulcerative colitis. Br J Surg.

[34] Lovegrove RE, Tilney HS, Remzi FH, Nicholls RJ, Fazio VW, Tekkis PP. To divert or not to divert a retrospective analysis of variables that influence ileostomy omission in ileal pouch surgery. Arch Surg Chic Ill 1960. 2011;146(1):82–8. 10.1001/archsurg.2010.304 21242450

[35] Larsen SG, Pfeffer F, Kørner H, Norwegian Colorectal Cancer Group (2019). Norwegian moratorium on transanal total mesorectal excision. Br J Surg..

[36] Coffey JC, Dillon MF, O’Driscoll JS, Faul E (2016). Transanal total mesocolic excision (taTME) as part of ileoanal pouch formation in ulcerative colitis—first report of a case. Int J Colorectal Dis.

[37] Hanke LI, Bartsch F, Försch S, Heid F, Lang H, Kneist W (2017). Transanal total mesorectal excision for restorative coloproctectomy in an obese high-risk patient with colitis-associated carcinoma. Minim Invasive Ther Allied Technol MITAT.

[38] Warusavitarne J, Kotze PG (2017). Double single-port transanal pouch surgery: a novel technique for rectal excision and ileo-anal pouch anastomosis for ulcerative colitis. J Coloproctology.

[39] Spinelli A, Cantore F, Kotze PG, David G, Sacchi M, Carvello M (2017). Fluorescence angiography during transanal trans-stomal proctectomy and ileal pouch anal anastomosis: a video vignette. Colorectal Dis..

[40] Carvello M, David G, Sacchi M, Di Candido F, Spinelli A (2018). Restorative proctocolectomy and ileal pouch-anal anastomosis for right-sided colonic adenocarcinoma in familial adenomatous polyposis: an abdominal laparoscopic approach combined with transanal total mesorectal excision—a video vignette. Colorectal Dis.

[41] Zaghiyan K, Warusavitarne J, Spinelli A, Chandrasinghe P, Di Candido F, Fleshner P (2018). Technical variations and feasibility of transanal ileal pouch-anal anastomosis for ulcerative colitis and inflammatory bowel disease unclassified across continents. Tech Coloproctology.

[42] Bjoern MX, Nielsen S, Perdawood SK (2019). Quality of life after surgery for rectal cancer: a comparison of functional outcomes after transanal and laparoscopic approaches. J Gastrointest Surg.

[43] Veltcamp Helbach M, Koedam TWA, Knol JJ, Velthuis S, Bonjer HJ, Tuynman JB (2019). Quality of life after rectal cancer surgery: differences between laparoscopic and transanal total mesorectal excision. Surg Endosc.

[44] Roumen RM, Rahusen FT, Wijnen MH, Croiset van Uchelen FA (2000). “Dog ear” formation after double-stapled low anterior resection as a risk factor for anastomotic disruption. Dis Colon Rectum..

[45] Gentilini L, Coscia M, Lombardi PM, Tanzanu M, Laureti S, Podda M (2016). Ileal pouch-anal anastomosis 20 years later: is it still a good surgical option for patients with ulcerative colitis?. Int J Colorectal Dis.

[46] Wiegel M, Meston C, Rosen R (2005). The female sexual function index (FSFI): cross-validation and development of clinical cutoff scores. J Sex Marital Ther.

[47] Vickers AJ, Tin AL, Singh K, Dunn RL, Mulhall J (2020). Updating the international index of erectile function: evaluation of a large clinical data set. J Sex Med.

[48] Andersson T, Lunde OC, Johnson E, Moum T, Nesbakken A (2011). Long-term functional outcome and quality of life after restorative proctocolectomy with ileo-anal anastomosis for colitis. Colorectal Dis.

